# Macrophage Activation in Follicular Conjunctivitis during the COVID-19 Pandemic

**DOI:** 10.3390/microorganisms11092198

**Published:** 2023-08-31

**Authors:** Carla Enrica Gallenga, Martina Maritati, Marco Mura, Francesco Di Virgilio, Pio Conti, Carlo Contini

**Affiliations:** 1 Department of Medical Sciences Doctoral Course Molecular Medicine, University of Ferrara, 44121 Ferrara, Italy; francesco.divirgilio@unife.it; 2 Ophthalmology Unit, University—Hospital Cona, 44124 Ferrara, Italy; marco.mura@unife.it; 3 Bone and Infections Lab, Santa Maria Maddalena NH, Occhiobello, 45030 Rovigo, Italy; mrtmtn#@unife.it; 4 Department of Medical Sciences, Infectious Diseases Unit, University of Ferrara, 44121 Ferrara, Italy; 5 Department of Translational Medicine and for the Romagna, Ophthalmology, University of Ferrara, 44121 Ferrara, Italy; 6 Department of Medical Sciences, University of Ferrara, 44121 Ferrara, Italy; 7 School of Medicine, Immunology, G. D’Annunzio University, Chieti-Pescara, 66100 Chieti, Italy; pioconti@yahoo.it; 8 Molecular Pharmacology and Drug Discovery Laboratory, School of Medicine, Tufts University, Boston, MA 02111, USA; 9 Department of Medical Sciences, Section Dermatology and Infectious Diseases, University of Ferrara, 44124 Ferrara, Italy; carlo.contini@unife.it

**Keywords:** COVID-19, ACE2&TMPRSS2, P2X7R, macrophages and activated T cells, follicular conjunctivitis

## Abstract

Among the symptoms of SARS-CoV-2, follicular conjunctivitis has become relevant. The conjunctiva acts as an open lymph node, reacting to the viral antigen that binds the epithelial cells, forming follicles of B cells with activated T cells and NK cells on its surface, which, in turn, talk to monocyte-derived inflammatory infected macrophages. Here, the NLRP3 inflammasome is a major driver in releasing pro-inflammatory factors such as IL-6 and caspase-1, leading to follicular conjunctivitis and bulbar congestion, even as isolated signs in the ‘asymptomatic’ patient.

## 1. Opinion

SARS-CoV-2 is inhaled as droplets or through surface contact and can cause COVID-19. The virus adheres to the angiotensin-converting enzyme 2 (ACE2) receptor, diffusely expressed in human eye surface epithelial cells, in adnexal glands and goblet cells. The trans-membrane serine protease 2 (TMPRSS2) is used for S protein priming by host cell protease [1], as demonstrated in SARS-CoV-2-infected TMPRSS2 knock-out mice that showed no pulmonary disease and lower viral replication [2]. TMPRSS2 proteolitically processes the Spike (S) viral protein, co-localizes with ACE2 at the cell membranes, and is the dominant driver of S protein activation [3]. The two protrusions of the N-peptidase domain of ACE2 provide a peptide substrate-binding site between them, where the extended SARS-CoV-2 receptor-binding domain (RBD) matches with the bottom side of the ACE2 small bump [4], while the N terminal helix of ACE2 accommodates in the outer surface recess of the receptor-binding motif (RBM). The expression is weaker in the cell membrane than in the cytoplasm, where the NLRP3 inflammasome could be activated by the P2X7 receptor (P2X7R), a plasma membrane receptor gated by extracellular adenosine triphosphate (ATP) [5], acting as a major driver in releasing pro-inflammatory factors such as IL-6 [6] and caspase-1.

After infecting monocytes, the hyperactivation of macrophages paves the way for hyper-inflammation in COVID-19 [7]. Activated T cells stimulate macrophages through tumor necrosis factor (TNF) and interferon gamma (IFNγ), and natural killer (NK) cells through IFNγ and GM–CSF receptors.

Furthemore, P2X7R has been suggested to bridge coagulation, releasing microvesicle-associated tissue factor (TF) and inducing a heightened pro-thrombotic response [8]. Microthrombi are also caused by tromboxane-A2 (TxA2) induction enhanced by IL-1β [9].

**Figure 1:**
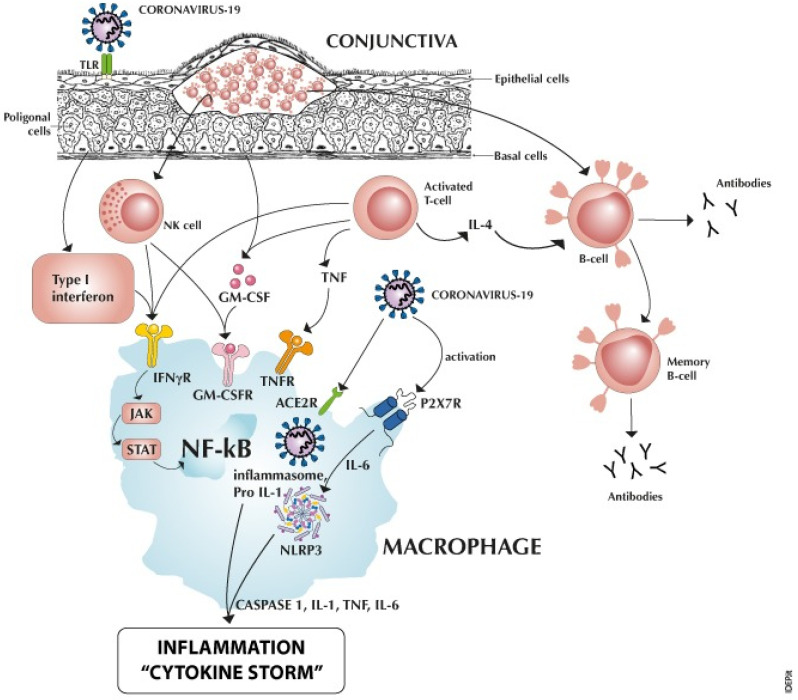
Follicular conjunctivitis induced by epithelial coronavirus infection.

As for the general concept, an antigen can be recognized as a non-self constituent and activates lymphocytes only if it somehow distorts or modifies the configuration of macrophage self-antigen [10,11]. According to Oppenheim [12] and LeBien and Tedder [13] lymphocyte activating factor (LAF) or interleukin 1 (IL-1), a single polypeptide chain produced by the macrophage cell lines, promotes antibody production by macrophage-depleted B lymphocytes to T cell-dependent antigens (Figure 1).

Figure 1 summarizes the cross-talks between the B lymphocytes in the conjunctival follicles and their macrophages. Mast cells are activated via plasma cell (not reported).

Macrophage activation and releases can induce a “cytokine storm” in lung alveolar cells and dwell in the conjunctival follicles of B lymphocytes.

Surface TLR (toll-like receptor) ACE2R (angiotensin-converting enzyme 2 receptor) binding the SARS-CoV-2 receptor-binding domain gives rise to follicles of activated Tcells releasing type I interferon, TNF (tumor necrosis factor), and the monomeric glycoprotein GM-CSF (granulocyte-macrophage colony-stimulating factor), while the coronavirus itself enters macrophages through ACE2R and P2X7R (purinergic receptor) [14,15]. High extracellular adenosine triphosphate (ATP) levels—inexistent in physiological conditions, but reaching high concentrations when released from immune cells in response to a tissue insult—are required for the receptor to be triggered and contribute to its role in cell damage signaling, activating the NLRP3 (NOD-like receptor P3) inflammasome through IL-6, which induces caspase-1 activation, leading to IL-1, IL-6, TNF, and caspase-1 maturation and release, and stimulating inflammation to create “cytokine storm” [9,16] and bulbar congestion. NF-kB (nuclear factor-kappa B) is a nuclear transcription factor present in all cytokine-producing cells. NK (natural killer) cells, large granular lymphocytes, talk with macrophages through the GM-CSF and IFN-γ (interferon-gamma) receptors, activating the JAK-STAT (Janus kinase/signal transducer and activator of transcription proteins) pathway that communicates information from outside of the cell to the nucleus, resulting in the activation of genes through the transcription process [7]. Interactions between B cell, macrophage, plasmacells and mastcells: B cells can differentiate into plasma cells and memory B cells under the stimulation of IL-4 from Th cells. Plasma cells continuously secrete immunoglobulins, which directly produce inflammation. Specifically, IgE can activate macrophage polarization and mast cell degranulation and subsequently increase their production of proteases such as MMPs (metalloproteinases) and cathepsins. These factors work together in the pathogenesis of extracellular matrix degradation and are an example of immune cell interactions in conjunctival disease.

The acute inflammatory follicular response is mostly present in the tarsal and fornix conjunctiva, semilunar fold, and caruncle, while the bulbar conjunctiva shows a picture of hyperemia, edema, and lymphangiectasia with bulbar congestion and hitching. The secretion is serous, often scarce, but never purulent unless bacterial co-infection occurs, with abundant colorable mononuclear cells in the smear. There, electron microscopy (EM) and immunoelectron microscopy (IEM) allow the identification and characterization of viral particles [17], while PCR is superior to ELISA for sensitivity and accuracy in detecting infections [18], where the conjunctiva and cornea seem to be the ophthalmic structures most affected by viral infections, as previously summarized by Frezzotti and Guerra [19], Sen et al. [20], and McHang et al. [21]. A new discovery showed for the first time that in the tears of vaccinated COVID-19 patients, ocular secretory IgA (sIgA) values are remarkably different vs. those of non-vaccinated patients [22], with significant differences in available vaccines. The IgA receptor (FcαR or CD89) can be found on the surface of neutrophils, eosinophils, monocytes, some macrophages, and dendritic cells [23].

Moreover, it is also interesting that a soluble form of the P2X7 receptor acts as an indicator of ocular inflammatory status, as has recently been documented in a PhD thesis in molecular medicine [24], which defined the presence and role of the soluble form (sP2X7) in normal and pathological human aqueous and vitreous humor. It can also be hypothesized to have a future significance for the liquid biopsy of intraocular tumors and for diabetic retinopathy. Therein, the hyperglycemia-induced damage to retinal pericytes leads to cell lysis, accompanied by the release of ATP into the extracellular environment, which in turn binds P2X7R on neighboring cells, activating the inflammasome and taking on the function of an inflammatory damage-signaling device via an autocrine/paracrine mechanism [25], resulting in a powerful trigger for vascular endothelial growth factor (VEGF) release, as described in the monocyte and macrophage activation pathway [7,26].

### Brief Commentary on the Histopathology and Clinical Behavior of Follicles in Comparison to Conjunctival Papillae

**Figure 2:**
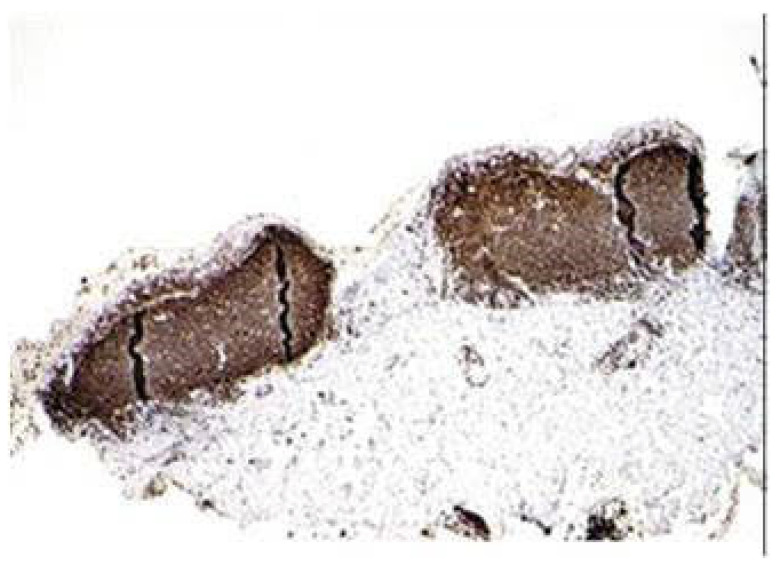
Section showing a reactive lymphoid follicle case stained immunohistochemically with a Bcell marker, CD20. This shows that the follicles are composed of B cells and have a non-destructive, well-defined architecture [27] (Courtesy Dr. S. Honavar, IJO editor-in-chief).

The cause of follicular conjunctivitis includes viral infection, chlamydial infection, topical drug-induced, Parinaud oculo-glandular disease, and idiopathic. It comprises nodules of lymphocytes with reactive germinal centers, composed of immature large Bcells, surrounded by a mantle of smaller mature Bcells (Figure 1). These nodules are present in the substantia propria and cause a smooth bulge of the overlying conjunctival epithelium. Haematoxylin and eosin (H&E)-stained reactive lymphoid follicles show tingible-body macrophages among the lymphocytes. Dendritic cells are represented. These macrophages tend to be a feature of a benign lymphoid follicle. Follicular conjunctivitis, stained immunohistochemically with the Bcell marker CD20 (CD20 is a molecule specific to mature B cells that works as a membrane-incorporated Ca^2+^ channel, (Figure 2), shows follicles composed of B cells [27].

Follicles must be differentiated with papillary conjunctivitis. Papillary conjunctivitis causes include allergic/atopic (vernal, seasonal, or perennial), topical drugs or preparation (even cosmetics), and chronic irritation (mechanical i.e., contact lens), or ocular diseases (dry eyes, superior limbic conjunctivitis) that induce the polygonal distortion of the epithelium. Each elevation is usually polygonal, larger than a follicle, and contains vertically orientated vessels around which are many inflammatory cells. The nature of the inflammatory cells can suggest the etiology. For example, if mast cells and eosinophils are seen, it points to an allergic/atopic etiology [27,28]. They comprise a fibrovascular core with a variety of inflammatory cells, and the surface is often covered in metaplastic squamous epithelium.

The papillae in allergic-type disorders are often packed full of eosinophils and mastcells (MC). Pro-inflammatory and anti-inflammatory cytokines play a key role in MC activation by neuropeptides. In the brain, they are activated by neuropeptide substance P (SP), corticotropin-releasing hormone (CRH), and neurotensin. Lauritano and coll. [29] suggest a therapeutic effect of the anti-inflammatory cytokines IL-37 and IL-38.

The practice of conjunctival biopsy in these inflammatory or allergic/immunological forms is rarely necessary; in cases of systemic immunopathology (i.e., Sjogren, sarcoidosis) it is customary to preferentially resort to a biopsy of the buccal mucosa. The conjunctival smear technique for the immunohistochemical evaluation of allergic forms is useful in research studies, but little practiced in clinical routine [30].

Moreover, dry eye-associated symptoms are frequently present in patients affected by allergic conjunctivitis. By performing qualitative and quantitative tests on tears, obtained from the inferior fornix, the immune activation state can be detected. Immunocytochemical markers for CD45RO, CD8, CD20, and EG2 (monoclonal antibody-binding eosinophil cationic protein) evaluated semi-quantitatively were found to be altered in our previous research [28]. They were reduced in allergic patients in comparison to the control group (*p* < 0.001). In conjunctival biopsies of allergic patients, a very high number of CD45RO+ and EG2+ cells was found (*p* < 0.001): a lower number of CD45RO+ cells and no EG2+ cells have been identified in control biopsies. Multivariate analysis showed a significant relationship between tear tests and conjunctival infiltrate (CD45RO+ and EG2+). The tear film alterations are strictly related to conjunctival immune infiltration. In particular, the reduction of the mucin-related component of tear film can be related to the toxic effect of the granule cationic proteins released by conjunctival activated eosinophils (EG2+ cells) [28].

During the development of experimental allergic conjunctivitis, conjunctival macrophages act as antigen-presenting cells (APC), that take up, process, and present antigens to T cells [31].

## 2. Conclusions

The complexity of immune cell interactions, their cross-talk, and the role of the cytokine microenvironment in the immune response are still under investigation. Considering the range of receptors expressed and the ability to produce cytokines that can both initiate and regulate inflammation, is possible to assume that the epithelium is central to immunity, with characteristics that bridge both innate and adaptive immune responses [11,32,33].

SARS-CoV-2 receptors allow infected macrophages to play a key role in the local conjunctiva (eye setting), oropharingeal tract (nose/throat setting)—as we previously reported [34]—and alveolar epithelial cells, until the appearance of a “cytokine storm”, as in Figure 1. It seems significant to signal the mechanisms of cellular cross-talk and the possibility that an acute, non-remitting microfollicular conjunctivitis presents itself as the only sign of an ‘asymptomatic’ but contagious SARS-CoV-2 viral infection [35]. Clinicians should suspect COVID-19-related follicular conjunctivitis from patients’medical history, absence of previous history of seasonal conjunctivitis, recurrent, relapsing, endemic COVID-19 clusters, or from a positive COVID-19 test (positive antigenic oral swab taken at the time of admission, molecular swab positivity, or conjunctival swab, according to Scalinci, Sarma, and Azzolini) [35,36,37].

Healthcare professionals nowadays are still facing an unprecedented global health issue which is affecting each medical specialty, requiring a holistic vision to exert the maximum effort to reduce the contagion rate and to treat patients to the best of their abilities, keeping the alert level high, despite fact that the WHO has declared an end to the pandemic.

## References

[1] Hoffmann M., Kleine-Weber H., Schroeder S., Krüger N., Herrler T., Erichsen S., Schiergens T.S., Herrler G., Wu N.-H., Nitsche A. (2020). SARS-CoV-2 Cell Entry Depends on ACE2 and TMPRSS2 and Is Blocked by a Clinically Proven Protease Inhibitor. Cell.

[2] Iwata-Yoshikawa N., Okamura T., Shimizu Y., Hasegawa H., Takeda M., Nagata N. (2019). TMPRSS2 Contributes to Virus Spread and Immunopathology in the Airways of Murine Models after Coronavirus Infection. J. Virol..

[3] Fraser B.J., Beldar S., Seitova A., Hutchinson A., Mannar D., Li Y., Kwon D., Tan R., Wilson R.P., Leopold K. (2022). Structure and activity of human TMPRSS2 protease implicated in SARS-CoV2 activation. Nat. Chem. Biol..

[4] Yin X., Zhang J. (2020). Advances in the research of ocular surface β gene coronavirus receptor. Chin. J. Experim. Ophthalmol..

[5] Cicko S., Köhler T.C., Ayata C.K., Müller T., Ehrat N., Meyer A., Hossfeld M., Zech A., Di Virgilio F., Idzko M. (2018). Extracellular ATP is a danger signal activating P2X7 receptor in a LPS mediated inflammation (ARDS/ALI). Oncotarget.

[6] Di Virgilio F., Dal Ben D., Sarti A.C., Giuliani A.L., Falzoni S. (2017). The P2X7 Receptor in Infection and Inflammation. Immunity.

[7] Merad M., Martin J.C. (2020). Pathological inflammation in patients with COVID-19: A key role for monocytes and macrophages. Nat. Rev. Immunol..

[8] Baroni M., Pizzirani C., Pinotti M., Ferrari D., Adinolfi E., Calzavarini S., Caruso P., Bernardi F., Di Virgilio F. (2007). Stimulation of P2 (P2X_7_) receptors in human dendritic cells induces the release of tissue factor-bearing microparticles. FASEB J..

[9] Conti P., Caraffa A., Gallenga C.E., Ross R., Kritas S.K., Frydas I., Younes A., Di Emidio P., Ronconi G., Toniato E. (2020). IL-1 induces throboxane-A2 (TxA2) in COVID-19 causing inflammation and micro-thrombi: Inhibitory effect of the IL-1 receptor antagonist (IL-1Ra). J. Biol. Regul. Homeost. Agents.

[10] Oppenheim J.J., Yang D. (2005). Alarmins: Chemotactic activators of immune responses. Curr. Opin. Immunol..

[11] Arnold C.E., Gordon P., Barker R.N., Wilson H.M. (2015). The activation status of human macrophages presenting antigen determines the efficiency of Th17 responses. Immunobiology.

[12] Oppenheim J.J. (1986). There is more than one interleukin 1. Immunol. Today.

[13] LeBien T.W., Tedder T.F. (2008). B lymphocytes: How they develop and function. Blood.

[14] Yuan Z., Lu Y., Wei J., Wu J., Yang J., Cai Z. (2021). Inflammatory Cells in AAA. Front. Immunol..

[15] Di Virgilio F., Tang Y., Sarti A.C., Rossato M. (2020). A rationale for targeting the P2X7 receptor in Coronavirus disease 19 (COVID-19). Br. J. Pharmacol..

[16] Conti P., Ronconi G., Caraffa A., Gallenga C.E., Ross R., Frydas I., Kritas S.K. (2020). Induction of pro-inflammatory cytokines (IL-1 and IL-6) and lung inflammation by Coronavirus-19 (CoV-19 or SARS-CoV-2): Anti-inflammatory strategies. J. Biol. Regul. Homeost. Agents.

[17] Erman A., Wechtersbach K., Velkavrh D., Pleško J., Frelih M., Kojc N. (2021). Just Seeing Is Not Enough for Believing: Immunolabelling as Indisputable Proof of SARS-CoV-2 Virions in Infected Tissue. Viruses.

[18] Gallenga P., Del Boccio M., Rapinese M., Di Iorio A., Toniato E., Martinotti S. (2011). Molecular Approach by PCR is the Best Method to Detect the Presence of Chlamydia Trachomatis and to Define the True Agent of Ocular Bacterial Inflammation. Int. J. Immunopathol. Pharmacol..

[19] Frezzotti R., Guerra R. (2006). OftalmologiaEssenziale.

[20] Honavar S., Sen M., Sharma N., Sachdev M. (2021). COVID-19 and Eye: A Review of Ophthalmic Manifestations of COVID-19. Indian J. Ophthalmol..

[21] McHarg M., Wang Y., Yakin M., Zeleny A., Caplash S., Sen H.N., Kodati S. (2022). Ocular symptoms in COVID-19 infection: A survey study. J. Ophthalmic Inflamm. Infect..

[22] Soffritti I., D’accolti M., Gallenga C.E., De Giorgio R., Guarino M., Maritati M., Bini F., Mazziga E., Contini C., Caselli E. (2023). Evaluation of Anti-SARS-CoV-2 IgA Response in Tears of Vaccinated COVID-19 Subjects. Viruses.

[23] de Freitas Santoro D., De Sousa L.B., Câmara N.O., De Freitas D., De Oliveira L.A. (2021). SARS-CoV-2 and ocular surface: From Physiology to Pathology, a route to understand transmission and disease. Front. Physiol..

[24] Gallenga C.E. (2023). Determination of the Soluble Form of the P2X7 Receptor in Acqueous Humour, Vitreous Humour and Serum under Normal and Pathological Conditions: sP2X7R as an Indicator of Ocular Inflammatory Status. Ph.D. Thesis.

[25] Platania C.B.M., Giurdanella G., Di Paola L., Leggio G.M., Drago F., Salomone S., Bucolo C. (2017). P2X7 receptor antagonism: Implications in diabetic retinopathy. Biochem. Pharmacol..

[26] Cho M., Hunt T.K., Hussain M.Z. (2001). Hydrogen peroxide stimulates macrophage vascular endothelial growth factor release. Am. J. Physiol. Circ. Physiol..

[27] Mudhar H.S. (2017). Update on conjunctival pathology. Indian J. Ophthalmol..

[28] Di Gioacchino M., Cavallucci E., Di Sciascio M.B., Di Stefano F., Verna N., Lobefalo L., Crudeli C., Volpe A.R., Angelucci D., Cuccurullo F. (2000). Increase in CD45RO+ Cells and Activated Eosinophils in Chronic Allergic Conjunctivitis. Immunobiology.

[29] Lauritano D., Mastrangelo F., D’Ovidio C., Ronconi G., Caraffa A., Gallenga C.E., Frydas I., Kritas S.K., Trimarchi M., Carinci F. (2023). Activation of mastcells by neuropeptides: The role of pro-inflammatory and anti-inflammatory cytokines. Int. J. Mol. Sci..

[30] Lobefalo L., D’Antonio E., Colangelo L., Della Loggia G., Di Gioacchino M., Angelucci D., Di Iorio A., Gallenga P.E. (1999). Dry Eye in Allergic Conjunctivitis: Role of Inflammatory Infiltrate. Int. J. Immunopathol. Pharmacol..

[31] Ishida W., Fukuda K., Kajisako M., Takahashi A., Sumi T., Van Rooijen N., Fukushima A. (2010). Conjunctival macrophages act as antigen-presenting cells in the conjunctiva during the development of experimental allergic conjunctivitis. Mol. Vis..

[32] Offiah I. (2011). Cross-Talk between Human T Cells, Mast Cells and Conjunctival Epithelial Cells. Ph.D. Thesis.

[33] Conti P. (2023). History of cytokines: My contribution. Eur. J. Neurodeg. Dis..

[34] Contini C., Gallenga C.E., Neri G., Maritati M., Conti P. (2020). A new pharmacological approach based on remdesivir aerosolized administration on SARS-CoV-2 pulmonary inflammation: A possible and rational therapeutic application. Med. Hypotheses.

[35] Scalinci S.Z., Battagliola E.T. (2020). Conjunctivitis can be the only presenting sign and symptom of COVID-19. IDCases.

[36] Sarma P., Kaur H., Kaur H., Bhattacharyya J., Prajapat M., Shekhar N., Avti P., Kumar S., MedhiMedhi B., Das D. (2020). Ocular Manifestations and Tear or Conjunctival Swab PCR Positivity for 2019-NCoV in Patients with COVID-19: A Systematic Review and Meta-Analysis. https://ssrn.com/abstract=3566161.

[37] Azzolini C., Donati S., Premi E., Baj A., Siracusa C., Genoni A., Grossi P.A., Azzi L., Sessa F., Dentali F. (2021). SARS-CoV-2 on Ocular Surfaces in a Cohort of Patients With COVID-19 From the Lombardy Region, Italy. JAMA Ophthalmol..

